# Participation in collaborative projects as a precursor of trust in organizations for individuals with intellectual disability

**DOI:** 10.1371/journal.pone.0242075

**Published:** 2020-11-10

**Authors:** Naiara Vidal, Vicente Martínez-Tur, Luminița Pătraș, Esther Gracia, Carolina Moliner, José Ramos

**Affiliations:** Research Institute in Personnel Psychology, Organizational Development and Quality of Working Life Research Unit in Work and Organizational Psychology (IDOCAL), University of Valencia, Valencia, Spain; Radboud University, NETHERLANDS

## Abstract

The present study focuses on organizations delivering services to individuals with intellectual disability, where trust relations between professionals and family members are required. More specifically, we examine the existence of significant differences in the degree to which family members and professionals trust each other. We also propose that their joint participation in collaborative teams (VI) will improve trust (VD). Specifically, our teams (experimental condition) designed and implemented collaborative projects with the participation of professionals and family members. Participants in the control condition did not participate in the collaborative projects. Our results confirmed that family members trust professionals more than professionals trust family members. Their joint participation in collaborative projects improved professionals’ trust in family members over time, compared to the control condition. The effect of collaborative projects was not significant for family members’ trust in professionals.

## Introduction

Trust within small groups is a relevant psychological mechanism to make possible the emergence and development of human social life [**1**]. This trust mechanism has been transferred to an artificial context designed by humans: the organization. In fact, trust within organizations and teams have become crucial for the adequate functioning of work relationships, impacting on performance and other relevant outcomes [2]. The present study focuses on organizations that provide services to individuals with intellectual disability, where fostering collaboration and trust between professionals and family members is especially relevant [3–9]. However, trust–defined in this context [10] as “the confidence that another person will act in a way to benefit or sustain the relationship”–(p. 6), is difficult due to the lack of opportunities for parents to collaborate with institutions [11].

Therefore, building trust between professionals and family members is an important challenge. Empirical evidence indicates that parents trust professionals more than professionals trust parents [10]. These authors interpreted this unbalanced trust in terms of differential positions of the actors. Professionals are experts and, thus, expected to be competent in treating individuals with intellectual disability. By contrast, family members do not have credentials that lead to high trust from professionals. This argument is congruent with social identity theory [12], which proposes that humans, as social beings, have a high capacity to classify themselves and others in different categories or groups using information from the social context [13, 14]. Status provides relevant input for this classification. In fact, members of high-status groups are more attractive and more highly evaluated [15]. Professionals have a status based on knowledge and expertise that provokes high trust from family members. This high trust is probably not reciprocated by professionals to the same extent. To capture this possible phenomenon, our first hypothesis is formulated to replicate the trust asymmetry found by Adams and Christenson [10]:

Hypothesis 1: Family members trust professionals more than professionals trust family members

The most relevant goal here is to propose and test an intervention that helps to improve trust. Scholars have called for investigation and implementation of collaboration as a way to improve trust between professionals and family members [5, 16]. This trust cannot be created by mandate; it requires interactions and collaboration over time [10]. However, there is a lack of empirical evidence testing interventions that assess the impact of collaboration. With this in mind, the current research study tests the impact of an intervention based on collaborative projects–where professionals, family members, and individuals with intellectual disability participate–on trust between professionals and family members over time. Based on social identity theory [12], collaborative projects are expected to remove *faultlines* between the actors and facilitate a context where all participants feel that they belong to the same group and have shared goals (e.g., improving quality of life of individuals with intellectual disability). Collaborative projects also allow participants to know each other. Family members have the opportunity to confirm professionals’ knowledge and skills directly. Professionals are forced to interact and view family members as active co-creators of the service who meaningfully contribute to shared goals. Considering all of these arguments, we propose the following hypothesis:

Hypothesis 2: The joint participation of family members and professionals in collaborative projects increases the trust between them

In summary, the present study investigates trust between professionals and family members in organizations delivering services to individual with intellectual disability. We were able to show that family members trust professionals more than professionals trust family members. In addition, their joint participation in collaborative projects improved professionals’ trust in family members over time.

## Materials and method

### Ethics statement

This study was conducted in accordance with the Declaration of Helsinki and it was evaluated and approved by the Ethical Committee of the University of Valencia. All participants were briefed about the objectives of the study and gave their written and informed consent on the experimental procedure where anonymity and confidentiality were guaranteed by the researchers. In addition, participants were informed that they were free to leave the study at any time or prevent the use of the data they provided. Below we describe how capacity to consent was determined for the participants in this study.

### Participants and procedure

The data were collected with the participation of 59 small centers whose goal is to achieve the social integration of individuals with intellectual and developmental disability. Participation of centers and individuals was confidential and voluntary. In each center, one autonomous team composed of two professionals and two families was created to design and implement a collaborative project (experimental condition). One individual with intellectual disability and one of his/her relatives (the one who had more frequent contact with the center) represented each family. In addition, we also considered a control condition with the participation of professionals and family members in each center. Members assigned to the control condition answered questions about trust, but they did not participate in teams. Professionals and families in the teams (experimental condition) and in the control condition were selected and distributed randomly. In each center, the research team trained an employee (contact employee), who was not a member of either the experimental or the control condition, to organize the process and collect data. Response rate was higher than 90% for both professionals and family members. Contact employees were also responsible for the distribution, explanation, and collection of signed informed consent forms in their centers, following the required ethical rules [17]. They explained the objectives and procedures of the research to all the participants in their centers. Regarding capacity to consent, these contact employees verified, during their conversations with participants, that the objectives, procedures, and voluntariness had been adequately understood before signing the informed consent and starting the research process.

In each center, one of the researchers gave an initial half-hour standardized speech to introduce the rationale for the project and the tasks to be performed. Only members of the teams (experimental condition) attended the speech. Each team should autonomously design and implement a project oriented toward the social integration–through social inclusion and self-determination–of the two individuals with intellectual disability on their team. Professionals and families should cooperate by attributing an active role to both actors and stimulating open communication, mutual respect for their skills, and shared objectives and decision-making. After the introductory speech, each team had two hours to design a project and create an action plan for the following eight weeks. The action plan had to be creative, original (never done before in the center), realistic, and able to be carried out in eight weeks. Each team had to establish specific actions and a timeline, involving all members (professionals and families) in the decision-making and in the implementation of the project. The researcher was not a member of the team and only acted as a facilitator. The autonomy of each team was fomented from the beginning. Team members had to interact and cooperate for eight weeks to implement the project. Professionals and family members of both the experimental and control conditions reported about trust before the meeting (T1) and four (T2) and eight (T3) weeks after the meeting.

Considering both experimental and control conditions, the baseline sample at T1 included 404 professionals (138 in the experimental condition and 266 in the control condition) and 457 family members (132 in the experimental condition and 325 in the control condition). Over time, a number of participants declined to participate in the two subsequent measures and some questionnaires had missing values. This sampling plan resulted in 329 professionals and 322 family members with usable surveys for all the three measurement times. Participants could decline to participate in T2 and T3 for different reasons (lack of time, maternity leaves, etc.). To check whether the final samples were biased, we compared them to individuals who participated in T1 but they did not participate in the complete data collection over time. Regarding professionals, there were no significant differences neither in their age (*F*_*(1*,*390)*_ = .15, *p >* .05) nor in the trust they had in family members (*F*_*(1*,*402)*_ = .17, *p >* .05). In addition, there were no significant differences in the distribution of men and women (χ^*2*^_*(1)*_ = 1.04, *p >* .05). We observed a similar pattern of results for family members with respect to distribution of men vs. women (χ^*2*^_*(1)*_ = .55, *p >* .05), age (*F*_*(1*,*447)*_ = .02, *p >* .05), and trust in professionals (*F*_*(1*,*451)*_ = .33, *p >* .05). These indicators showed that the final samples of the study were not biased.

The final samples in the experimental condition (participating in autonomous teams) were composed of 131 professionals and 118 family members. The final samples in the control condition were composed of 198 professionals and 204 family members. On average, family members were 56.79 years old (*SD* = 10.6), whereas professionals were 38.92 years old (*SD* = 9.19). In general, 75.5% of family members and 76% of professionals were women.

### Measures

At each measurement time (T1-T3), professionals reported on the degree to which they trust in family members. In the same way, family members reported their trust in the professionals. Our measure was based on the General Trust Scale developed by Butler [18]. Accordingly, three items referred to overall trust: “I trust the professionals/family members of this center”; “I feel I can trust the professionals/family members of this center”; and “I believe that the professionals/family members of this center are trustworthy”. Alpha coefficients were good for both professionals (.90; .92; and .90 for T1; T2; and T3, respectively) and family members (.89; .93; and .89 for T1; T2; and T3, respectively). All the items were scored on a 5-point rating scale with anchors of 1 (*Strongly disagree*) and 5 (*Strongly agree*).

## Results

### The trust asymmetry

**Fig 1:**
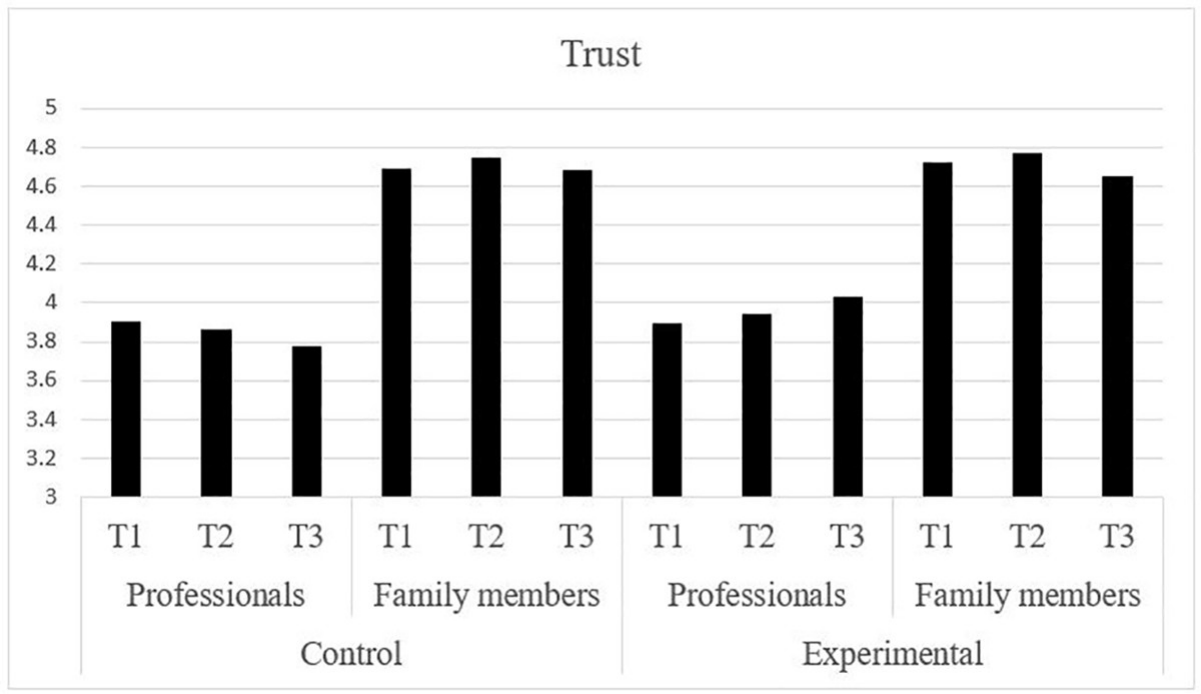
Trust of professionals and family members.

Our results supported Hypothesis 1. Our *t*-test analyses confirmed that trust was higher among family members than among professionals at the three measurements times and for both the experimental condition (T1—*t* = 10.14; *p* < .05; T2—*t* = 10.56; *p* < .05; T3—*t* = 7.83; *p* < .05) and the control one (T1—*t* = 12.49; *p* < .05; T2—*t* = 14.29; *p* < .05; T3—*t* = 13.79; *p* < .05). Therefore, family members trust professionals more than professionals trust family members (see Fig 1).

#### The effect of participation in collaborative projects

**Fig 2:**
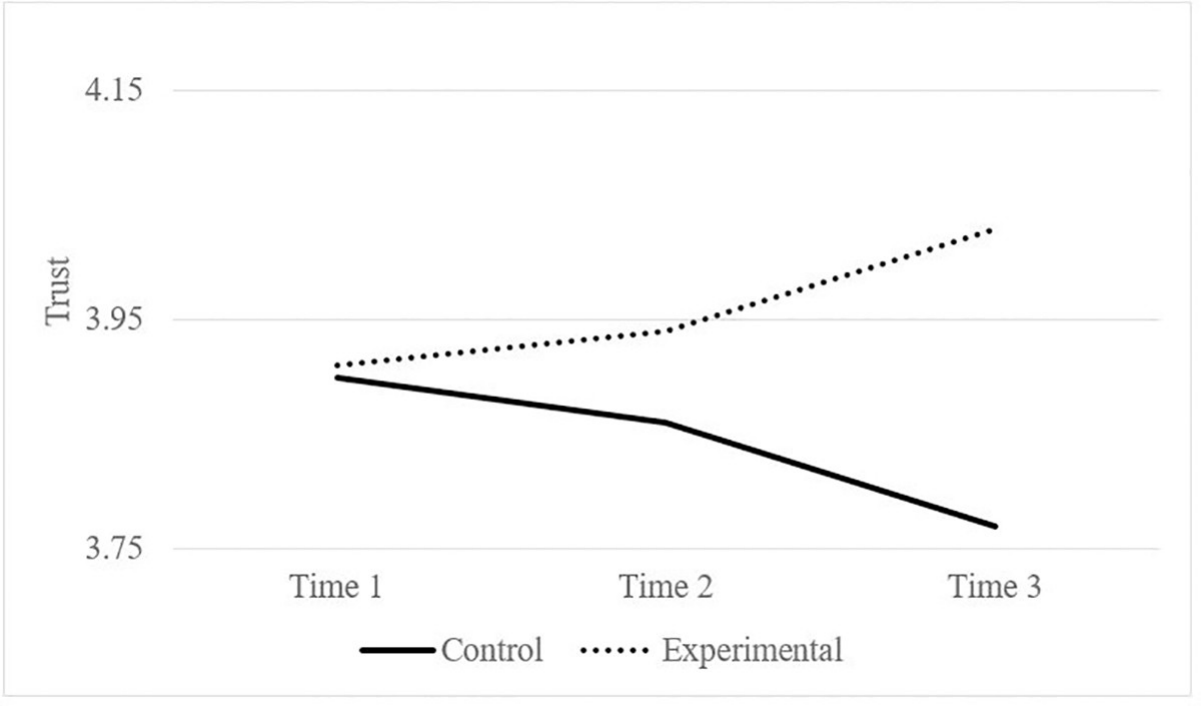
Trust of professionals in family members. Experimental vs. control conditions.

Our results supported Hypothesis 2, but only for professionals. We carried out two repeated-measures ANOVAs, one for professionals and the other for family members. Regarding professionals, neither time nor condition had significant direct effects on trust. However, as expected, the interaction between time (T1; T2; and T3) and condition (experimental vs. control) was statistically significant (*F*_*(2*,*642)*_ = 6.24, *p* < .05, *η =* .019). Participation in collaborative projects (experimental condition) improved professionals’ trust in family members over time, compared to professionals who did not participate in the projects (control condition) (see Fig 2). By contrast, the time and condition interaction was not statistically significant (*F*_(2,604)_ = 0.22, *p* = .79, *η* = .00) for family members.

Because professionals and family members pertained to different centers, it is possible that the impact of collaborative projects depends on center membership (e.g., some centers may have a more positive attitude towards cooperative projects than others). For this reason, we also computed a mixed-effect regression model with random intercept to test the difference in the rate of change in the trust score, over the three time points, controlling for center membership of family members and professionals. This allowed us a second test of our hypotheses where we controlled for the center membership of participants. Again, and supporting Hypothesis 1, type of participant (professionals vs. family members) was significant in predicting trust change, *b* = -.71, t (1859) = -8.60, *p* < .05, with family members showing higher levels of trust than professionals. Regarding Hypothesis 2, the interaction of time (T1; T2; and T3), condition (experimental vs. control), and type of participant (professionals vs. family members) was also statistically significant (*F*_*(1*,*1317)*_ = 8.32, *p* < .05). To break down the interaction, we rerun the analysis for each of the type of participant. The interaction of time and condition was not statistically significant (*F*_*(1*,*640)*_ = 0.65, *p* > .05) for family members. By contrast, participation in collaborative projects (experimental condition) improved trust of professionals in family members over time, compared with professionals who did not participate in the projects (control condition), *F*_*(1*,*658)*_ = 11.28, *p* < .05, with a significant difference in slope *b* = .13, t (658) = -3.36, *p* < .05. Therefore, these analyses provided additional support for the significant impact of collaborative projects on professionals’ trust in family members.

## Discussion

Our results consistently supported a trust unbalanced situation in organizations for individuals with intellectual disability, showing that family members trust professionals more than professionals trust family members. We also found that our collaborative intervention improves professionals’ trust in family members over time. However, the effect on family members was not significant. In the following paragraphs, we discuss the theoretical and practical implications of these results.

To understand trust between professionals and family members, one very informative result is the general level of trust corresponding to each actor. Family members’ scores are almost optimal, with values very close to the maximum possible. By contrast, although scores corresponding to professionals are quite high, they only exceed the value of four on the trust measure scale after eight weeks of cooperation with family members in the experimental condition. The lack of significant effects of collaborative projects on family members is probably due to the absence of real possibilities for improvement. The scores are so high from the beginning (before the intervention) that it is very difficult to further improve family members’ trust in professionals. This phenomenon is very similar to the “unconditional trust” in professionals that Angell et al. [19] observed in their interviews with mothers. Family members’ trust in professionals is strong and confirms the importance of credentials and reputation. Congruently with social identity theory [12], the high-status group (i.e., professionals) is positively evaluated [15], generating high levels of trust among family members.

Although the collaborative projects were organized and implemented for a limited time period (eight weeks), it was enough to cause a significant change, compared to professionals who did not participate in the teams. This indicates that joint cooperation through collaborative projects can be a promising way to reach optimum levels of professionals’ trust in family members, if these projects are generalized and have continuity. These results are consistent with the idea that collaboration provides opportunities for the professional to meet family members in their role as active co-creators of services, thus improving trust in them. This argument has been repeatedly formulated in the literature [3, 8, 16], but there is still a lack of research testing the effects of collaborative interventions on trust. Involving family members is not only the right thing to do, in terms of justice, but it is also a strategy that leads professionals to consider families as trustworthy and active co-creators of the service. To do so, some characteristics have to be considered when designing teams [20], such as the recognition of family members’ knowledge and skills, opening up channels of information and communication, agreement about goals, and shared planning and decision-making. In other words, the expert model of functioning, where the professional has most of the decision-making power, is replaced by a developmental approach where both families and professionals cooperate and contribute significantly to shared goals (i.e., improvement of quality of life of individuals with intellectual disability). Creating an alliance between professionals, family members, and individuals with intellectual disability could reinforce their bonds and establish successful partnerships in order to achieve shared goals based on trust.

As in all studies, the present study has limitations that could provide relevant inputs for future investigations. This research study was carried out within a specific type of organization: centers for individuals with intellectual disability. Although this type of service shares relevant characteristics with other organizations, testing the effects of collaborative projects in other contexts (e.g., hospitals, educational institutions) will help to examine the generalizability of our findings. Another limitation of the current research study is the lack of consideration of mechanisms underlying the dynamics of teams involved in the collaborative projects. The design of our research study followed principles that allow solid conclusions, including the comparison of experimental vs. control condition, the pre (T1) vs. post measurement (T2 and T3) of trust, and center membership control. Nevertheless, variability might exist between teams, for example, in the openness of the communication over time. Controlling for this variability could offer a richer view of the evolution of the collaboration between professionals and family members. Finally, the creation of teams and alliances with the participating professionals, family members, and individuals with intellectual disability can extend the positive effects beyond trust. For example, it could allow institutions to have an impact on the community by removing social prejudices related to intellectual disability and improving the rights of vulnerable groups as citizens of our societies.

Despite these limitations, the current research study contributes to knowledge and practice about trust between professionals and family members in two relevant ways. First, our findings confirm the distance between the two aforementioned actors in terms of trust: professionals express lower trust in family members than family members do in professionals. Second, we provide support for the argument that collaborative projects improve professionals’ trust in family members. Professionals’ trust in family members improves because they have the opportunity to interact with them in a meaningful way, that is, by viewing the family as an active actor capable of contributing to shared goals.

## Supporting information

S1 Dataset Family members. (XLSX)

S2 Dataset Professionals. (XLSX)
